# Supplementary material for: Identifying Function Determining Residues in Neuroimmune Semaphorin 4A
Source: Int J Mol Sci. 2022 Mar 11;23(6):3024. doi: 10.3390/ijms23063024 (PMC8953949; doi:10.3390/ijms23063024)
Supplement: Supplementary file 1 [file ijms-23-03024-s001.zip › ijms-1512316-supplementary.pdf]

Supplementary Materials:

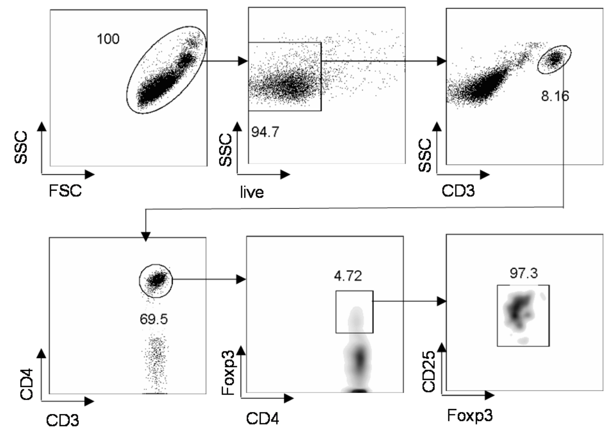

Supplemental Figure S1. Gating strategy for the analysis of human circulating Treg cells by FACS. Treg cells were assessed by flow cytometry using specific Abs to corresponding cell surface and intracellular molecules. Dead cells were eliminated from analysis based on their loss of membrane integrity and, thus, inclusion of a dead cell dye. Gated CD3 + CD4+ cells were further selected to evaluate the relative number of Treg cells based on the Foxp3 expression. Appropriate isotype-matching Ab was used as a control for Foxp3 staining. CD4 + Foxp3+ cells co-express CD25 marker.

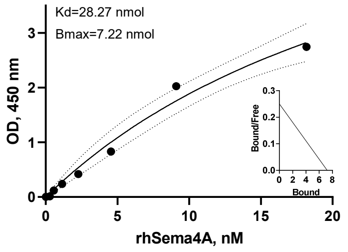

Supplemental Figure S2. Sema4A binds NRP1 with high affinity. Analysis of Sema4A binding to NRP1 by the direct ligand-receptor binding ELISA. Calculations of the equilibrium dissociation constant was performed utilizing GraphPrizm 8.0. software. For the Kd value determination, the data was fitted to the 'One site - specific binding with Hill slope'. The curves demonstrate a direct 1:1 ligand-receptor biding and are representative of three independent assays.

```
Sema4A 37   GPMPRVRYYAGDERRALSF--FHQKGLQDFDTLLLSGDGNTLYVGAREAILALDIQDPGV  94
             P+PR+ +    E R +    FH+  + ++  LLLS D +TLY+GAREA+ A++  +  +
Sema4D 25   APIPRITW----EHREVHLVQFHEPDIYNYSALLLSEDKDTLYIGAREAVFAVNALN--I  78

Sema4A 95   PRLKNMIPWPASDRKKSECAFKKKSNETQCFNFIRVLVSYNVTHLYTCGTFAFSPACTFI  154
               ++ + W  S+ KK++CA K KS +T+C N+IRVL   + T LY CGT AF PAC  +
Sema4D 79   SEKQHEVYWKVSEDKKAKCAEKGKSKQTECLNYIRVLQPLSATSLYVCGTNAFQPACDHL  138

Sema4A 155  ELQDSYLLPISEDKVMEGKGQSPFDPAHKHTAVLVDGMLYSGTMNNFLGSEPILMRTLGS  214
             L     L  +ED    GKG+ PFDPAH +T+V+VDG LYSGT  NFLGSEPI+ R
Sema4D 139  NLTSFKFLGKNED----GKGRCPFDPAHSYTSVMVDGELYSGTSYNFLGSEPIISRNSSH  194

Sema4A 215  QPVLKTDNFLRWLHHDASFVAA-----IPST-----QVVYFFFEETASEFDFFERLHTSR  264
             P L+T+  + WL+ + SFV A      P +       VYFFF E + E++F  R+   R
Sema4D 195  SP-LRTEYAIPWLN-EPSFVFADVIRKSPDSPDGEDDRVYFFFTEVSVEYEFVFRVLIPR  252

Sema4A 265  VARVCKNDVGGEKLLQKKWTTFLKAQLLCTQPGQ-LPFNVIRHAVLLPADSPTAPHIYAV  323
            +ARVCK D GG + LQKKWT+FLKA+L+C++P   L FNV+R   +L +     P  YA+
Sema4D 253  IARVCKGDQGGLRTLQKKWTSFLKARLICSRPDSGLVFNVLRDVFVLRSPGLKVPVFYAL  312

Sema4A 324  FTSQWQVGGTRSSAVCAFSLLDIERVFK-GKYKE---LNKETSRWTTYRGPETNPRPGSC  379
            FT Q    G   SAVCA++L   E VF  GKY +   + +  ++W  Y GP   PRPG+C
Sema4D 313  FTPQLNNVGL--SAVCAYNLSTAEEVFSHGKYMQSTTVEQSHTKWVRYNGPVPKPRPGAC  370

Sema4A 380  SVGPSS-----------DKALTFMKDHFLMDEQVV---GTPLLVKSGVEYTRLAVETAQG  425
                +            DK L F+KDH LMD+ V      P L+K  V YT++ V+  Q
Sema4D 371  IDSEARAANYTSSLNLPDKTLQFVKDHPLMDDSVTPIDNRPRLIKKDVNYTQIVVDRTQA  430

Sema4A 426  LDGHSHLVMYLGTTTGSLHKAVVSGDSSAHLVEEIQLFPDPEPVRNLQLAPTQGAVFV--  483
            LDG  + VM++ T  G+LHKA +S + + H++EE QLF D EPV+ L L+  +G  FV
Sema4D 431  LDGTVYDVMFVSTDRGALHKA-ISLEHAVHIIEETQLFQDFEPVQTLLLSSKKGNRFVYA  489

Sema4A 484  GFSGGVWRVPRANCSVYESCVDCVLARDPHCAWDPESRTCCLLSAPNLNSWKQDMERGNP  543
            G + GV + P A C  + +C DCVLARDP+CAW P + TC  L      S     E
Sema4D 490  GSNSGVVQAPLAFCGKHGTCEDCVLARDPYCAWSPPTATCVALHQTESPSRGLIQEMSGD  549

Sema4A 544  EWACASGPMSRSLRPQSRPQIIKEVLAVPNSILELPCPHLSALASYYWSHGPAAVPEASS  603
               C          P       ++         EL C   S LA  +W      V +A S
Sema4D 550  ASVC----------PDKSKGSYRQHFFKHGGTAELKCSQKSNLARVFWKFQ-NGVLKAES  598

Sema4A 604  TVY-----NGSLLLIVQDGVGGLYQCWATE  628
              Y        L+  + +G  G+YQC + E
Sema4D 599  PKYGLMGRKNLLIFNLSEGDSGVYQCLSEE  628
```

Supplemental Figure S3. Sequence alignment of sema domains representing human Sema4A and Sema4D molecules and highlighted in red residues in Sema4A critical for binding to Plexin B1. The corresponding sequences were aligned using BLAST. The sequence alignment demonstrated 40% homology between these two Class IV semaphorin members. Two Sema4A residues out of seven (see Figure 6) identified by BPPS-SIPRIS analysis as differentially conserved and appear to be near putative Plexin B1 receptor biding sites based on homology modeling are shown in red color. Methionine at the position 198 was replaced with alanine in Mu M198A Sema4A and phenylalanine at the position of 223 was replaced with alanine in Mu F223A Sema4A.

Supplemental Fig. S4, page 1

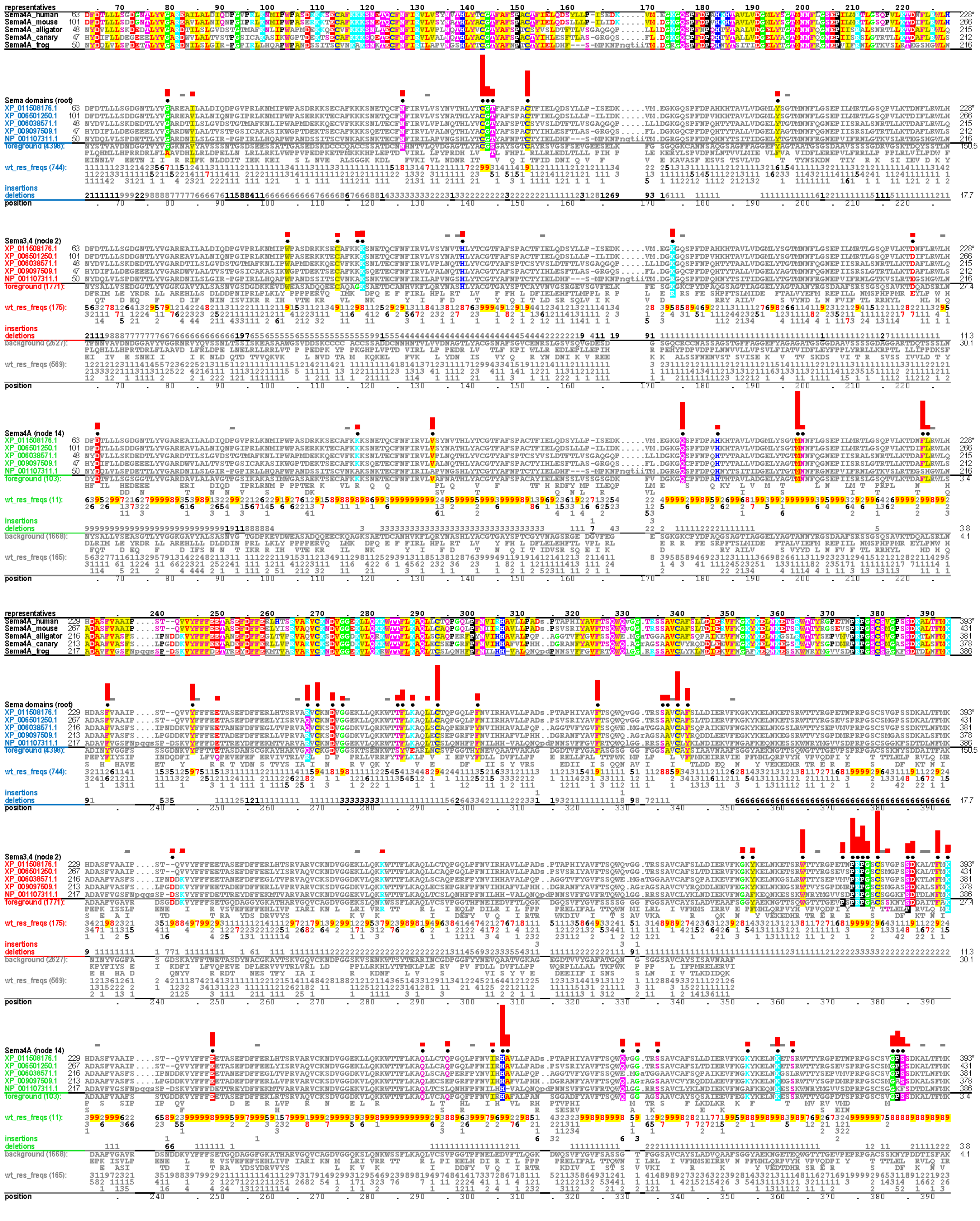

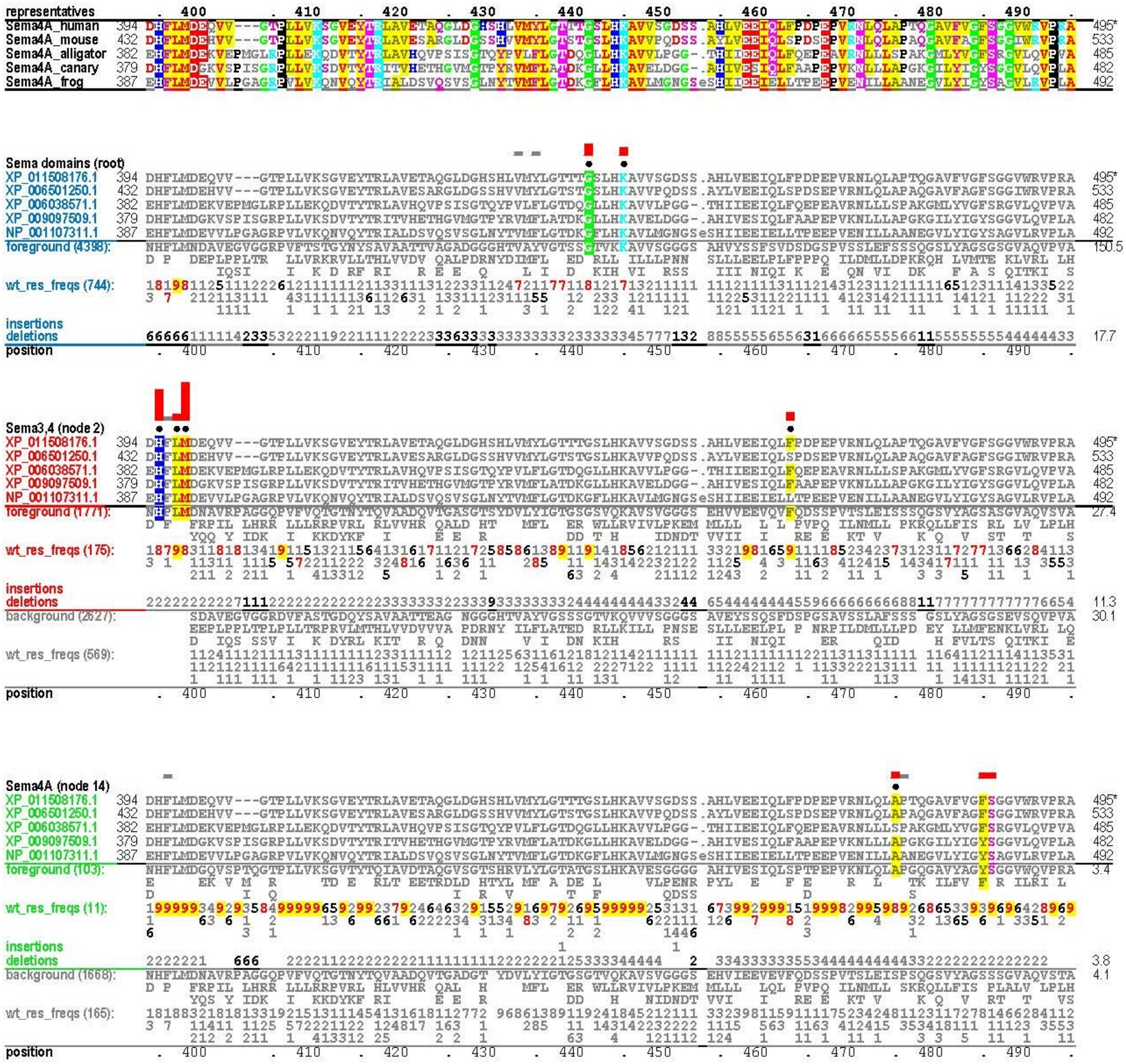

Supplemental Figure S4. BPPS generated contrast alignments showing the residues distinctive of subgroups along the lineage: Sema-domain superfamily (root), Sema3,4 family (node 2), and Sema4A subfamily (node 14). Shown in four distinct formats is an aligned set of 5 representative Sema4A domains from chordate taxa; these sequences are closely related to the first sequence, human Sema4A; their NCBI identifiers are shown for each subgroup. Aligned residues are highlighted to reveal conserved patterns that most distinguish each subgroup and to indicate amino acid biochemical properties based on the following color code: red font with yellow highlight, non-polar (AVILMWFY); blue font with yellow highlight, cysteine (C); red, acidic (DE); cyan, basic (KR); magenta, polar (STNQ); green, glycine (G); blue, histidine (H); black, proline (P). Non-distinguishing positions are shown in gray font. The red bars above highlighted columns quantify the selective pressures [based on a semi-logarithmic scale] shifting pattern residue positions away from other, closely related subgroups. Directly below the aligned sequences, the characteristic pattern residues at each position are shown and, directly below these, corresponding frequencies are given in integer tenths. A ‘7’, for example, indicates that the corresponding residue occurs in 70-80% of the foreground sequences. Below the main alignment, pattern residues characteristic of the next higher category (i.e., the background) are similarly indicated.

Supplemental Table S1. BPPS-defined sema domain node assignments.

node	subnode	sub-subnode	sema	taxa	# seqs
18			7A	chordates	121
8			1A,2A,5B	various metazoans	334
	16		5A,5B	chordates	165
6			6C,6D	"	181
	19		6A,6B	"	205
3			plexins	various metazoans	149
	23		A1-4,B,C1	non-chordate metazoans	177
	17		B2	chordates	156
	12		B1,B3	"	214
	7		MSPR	"	169
		21	HGFR	"	134
	5		A2, A4	"	199
		20	A1,A3	"	156
2			Sema3,4	"	333
	22		4C,4D	"	214
	14		4A	"	103
	10		4B	"	145
	9		4G	"	163
	4		Sema3	"	454
		13	3E	"	110
		15	3B	"	127
		11	3G	"	122

MSPR = macrophage-stimulating protein receptor
HGFR = hepatocyte growth factor receptor
